# Supplementary material for: Improved genotype inference reveals cis- and trans-driven variation in the loss-of-heterozygosity rates in yeast
Source: Genetics. 2025 Dec 22;232(4):iyaf274. doi: 10.1093/genetics/iyaf274 (PMC13050209; doi:10.1093/genetics/iyaf274)
Supplement: iyaf274_Supplementary_Data [file iyaf274_supplementary_data.zip › File_S1_-_Supplementary_Figures_GENETICS-2025-308599.pdf]

Supplementary Figures

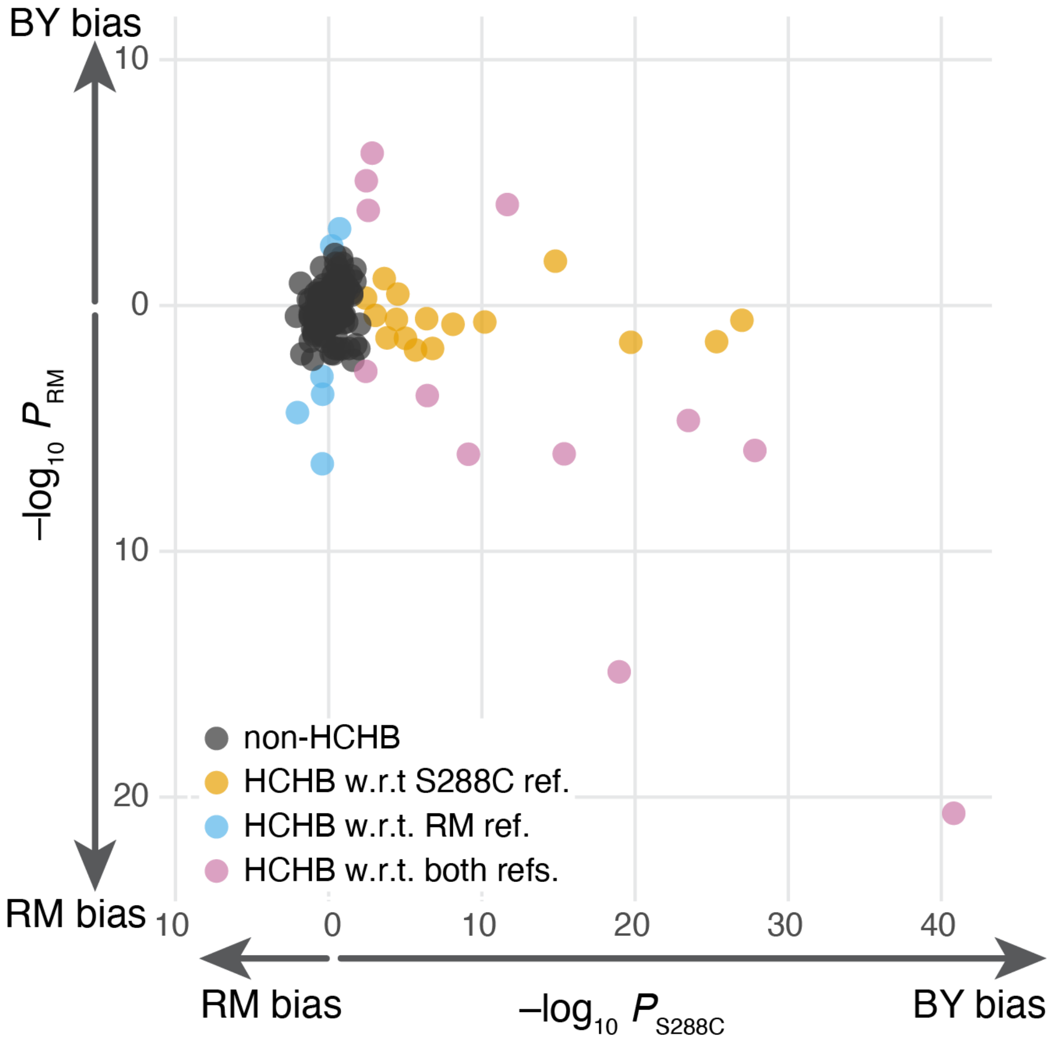

Figure S1. Homolog bias among LOH events is reference-sequence specific. Each point is a 50-kb window. *x*- and *y*-axes show the binomial *P*-value and homolog favored with respect to the S288C and RM references, respectively, as in Figure 1C. Windows identified as HCHB with respect to at least one reference are colored, as indicated in the legend. Non-HCHB windows are shown in grey.

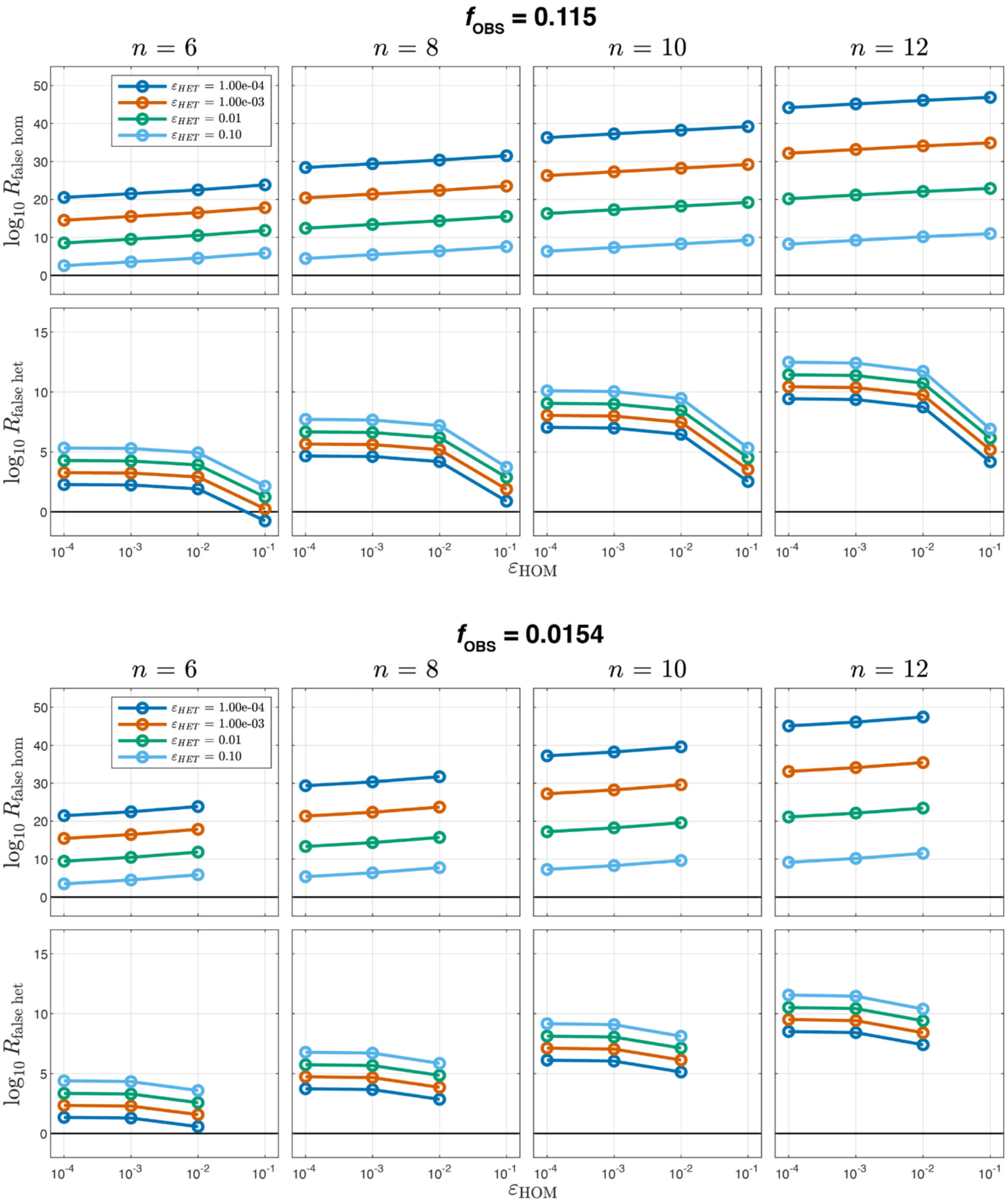

Figure S2. False homozygous and false heterozygous odds ratios. Each panel shows how the respective odds ratio depends on the false homozygote and false heterozygote error probabilities ε_{HOM} (x axis) and ε_{HET} (colors) for the configuration of end-point clones that is most discordant with respect to the ancestor (see text for details). Columns correspond to different numbers of genotyped end-point clones n, as indicated. Top panels show results for $f_{\text{OBS}} = 0.115$, bottom panels show results for $f_{\text{OBS}} = 0.0154$. Note that the bottom panels have only three points per line because of the constraint $\varepsilon_{\text{HOM}} \leq f_{\text{OBS}}$.

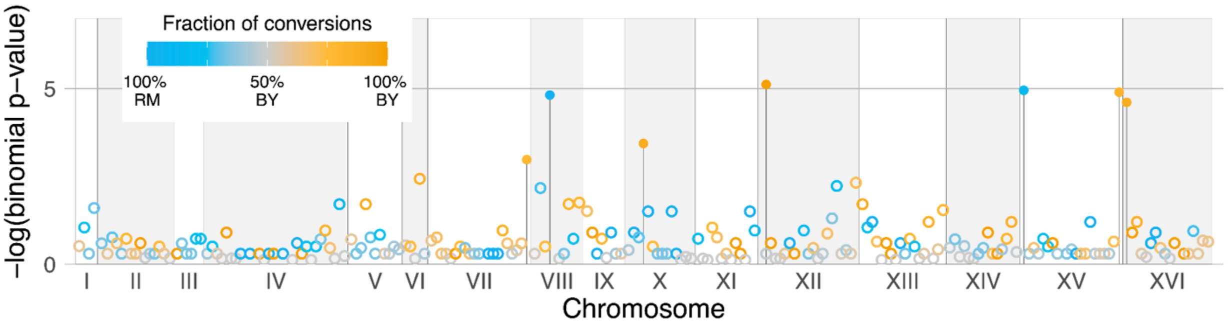

Figure S3. Distribution of HCHB windows across yeast genome after reference-symmetric genotyping. Same as Figure 3B but prior to heterozygous genotype filtering.

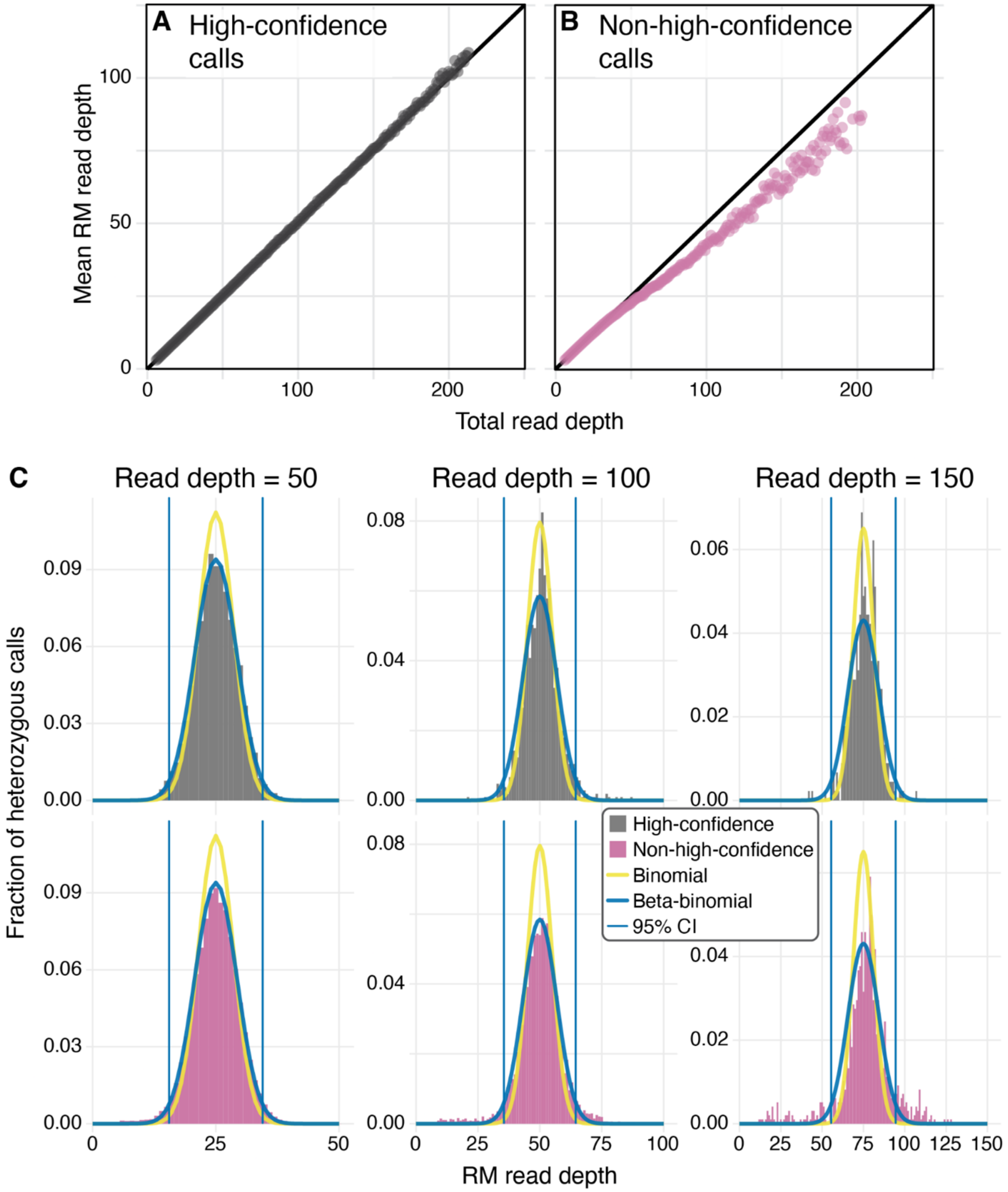

Figure S4. RM allele counts at high- and non-high-confidence heterozygous sites have distinct distributions. A. The mean RM allele count at high-confidence heterozygous sites across read depths (see Section "Heterozygous genotype filter" in Materials and Methods). **B.** Same as A but for non-high-confidence heterozygous sites. **C.** The distribution of RM allele counts for three representative read depths at high-confidence (grey bars) and non-high-confidence (red bars) heterozygous sites. Distributions at high-confidence sites are well described by the beta-binomial model (light blue), whereas the distributions at non-high-confidence sites have heavier tails. The beta-binomial 95% confidence intervals by which we reject dubious heterozygous calls are shown as dashed light blue lines.

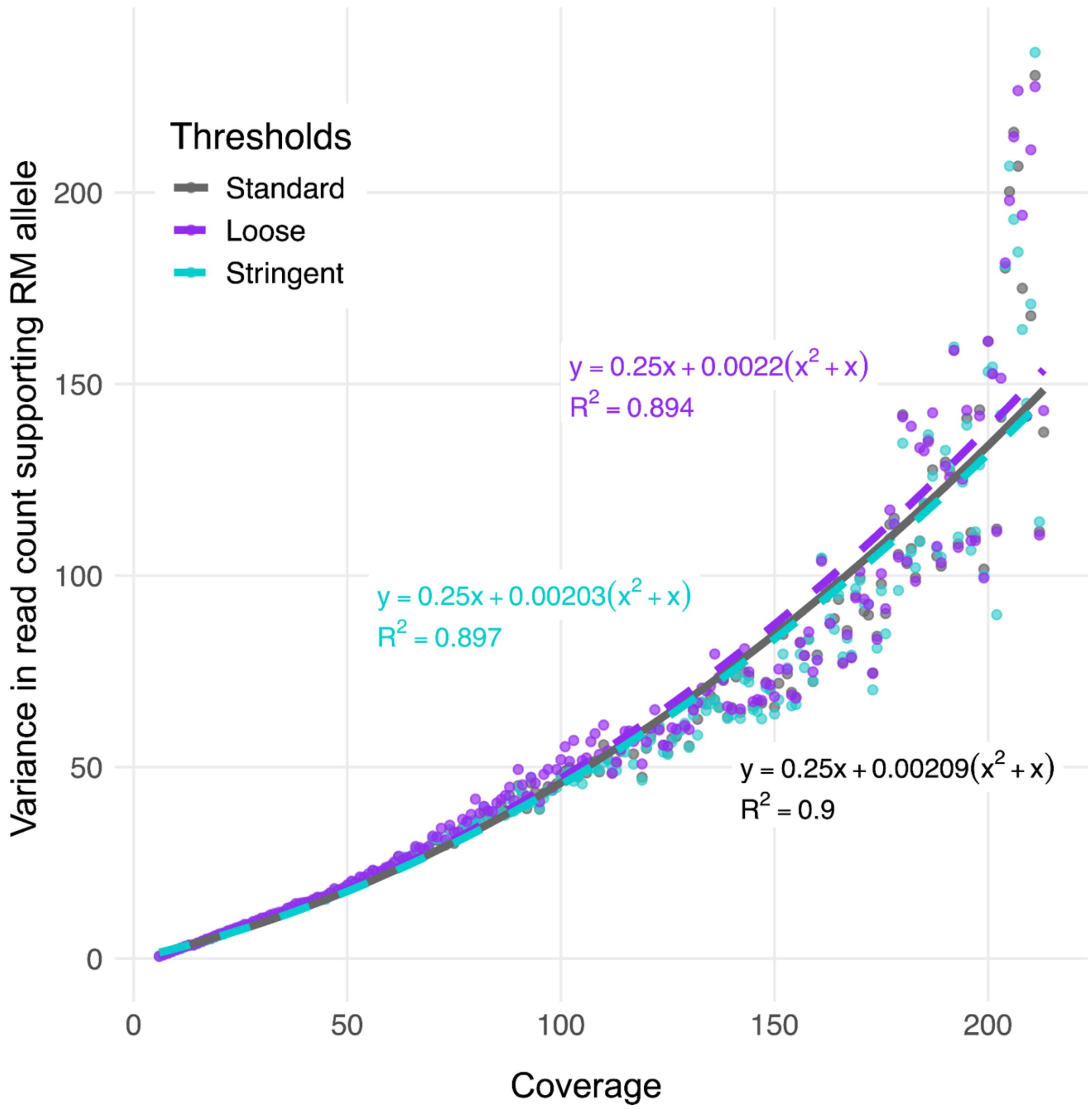

Figure S5. The heterozygous call filter is robust with respect to the definition of high-confidence sites. Same as gray points in Figure 3A but for different definitions of high-confidence heterozygous sites. Gray points represent the "standard" definition (15/16 of founders and 2/3 of end-point clones are called heterozygous at such sites; these are are the same points as in Figure 3A), blue points represent a "stringent" definition (all founders and 3/4 of end-point clones) and purple represents a "loose" definition (1/2 of founders and 1/2 of end-point clones).

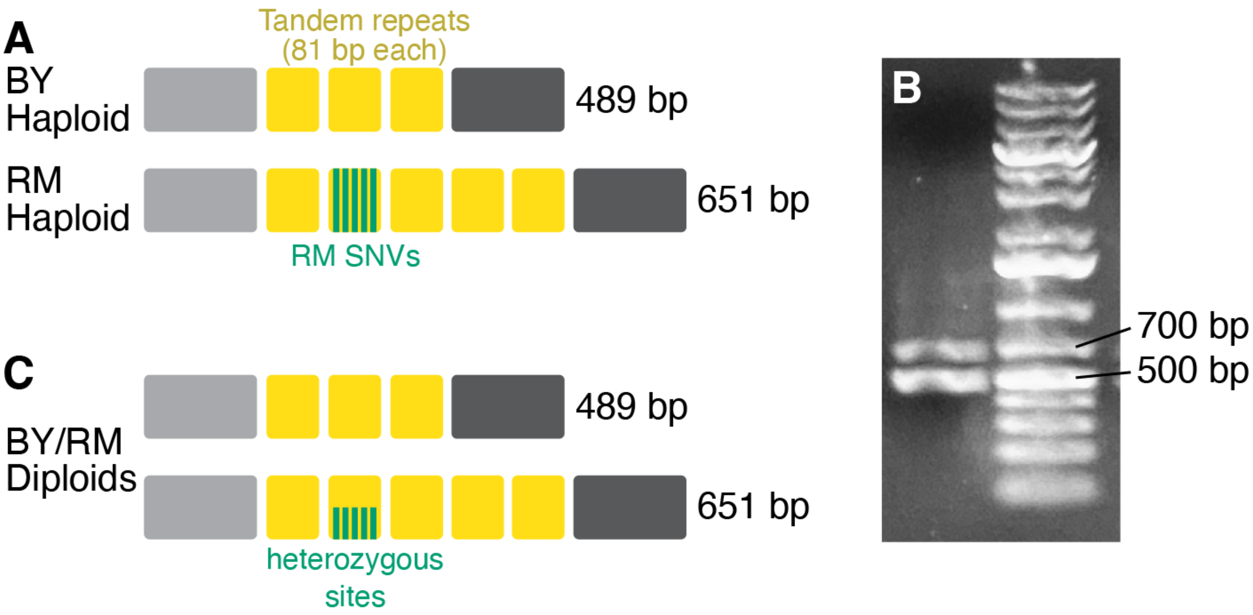

Figure S6. Structural variation at Chr. XII HCHB region confounds genotyping. A. We designed primers amplifying the region of Chr. XII that contains a structural variant between RM and BY genomes. The BY allele contains three 81 bp tandem repeats, the RM allele contains five of these repeats with one repeat carrying five single nucleotide variants (SNVs). **B.** The PCR amplification of this region in all of our diploid ancestor and end-point clones reveals two bands of expected lengths. **C.** Sanger sequencing of the two bands showed that the short fragment is identical to the BY reference, as expected, whereas the long fragment is heterozygous for the five SNVs, which is unexpected.

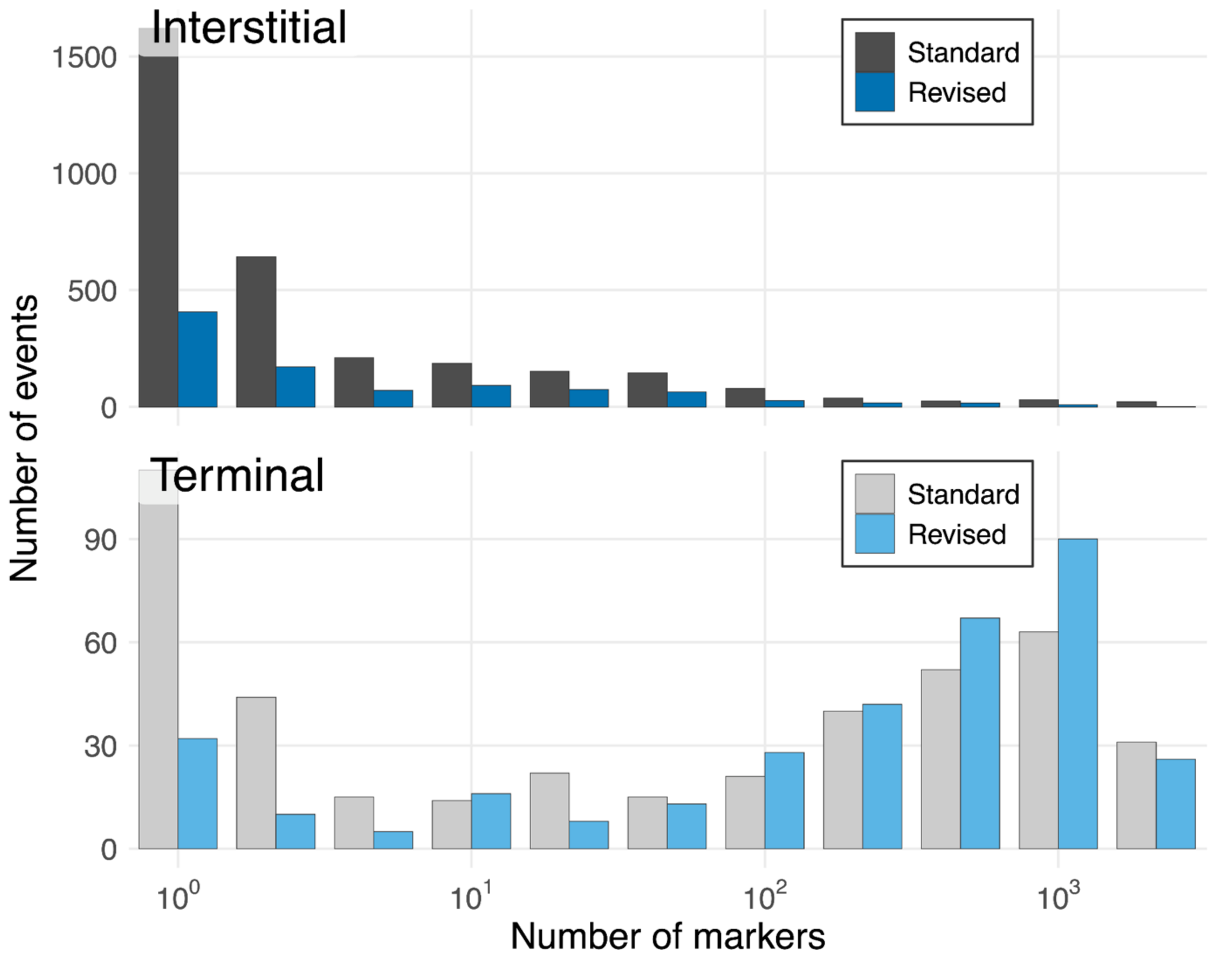

Figure S7. Distribution of LOH events supported by different numbers of markers under the standard and revised methods.

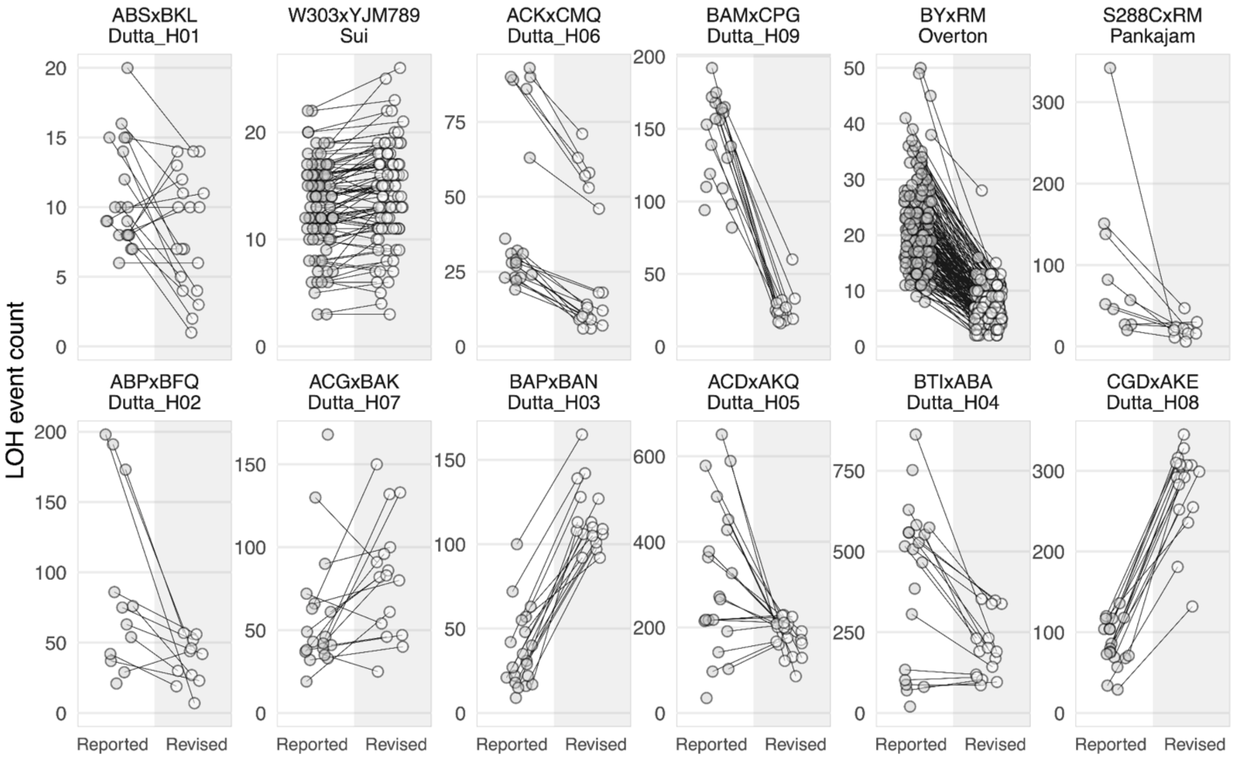

Figure S8. Comparison of previously published and revised LOH counts across yeast hybrids. Each end-point clone is represented as a pair of points connected by a line (reported on the left, revised using our method on the right). Revised counts are prior to the application of corrections for undetected events.

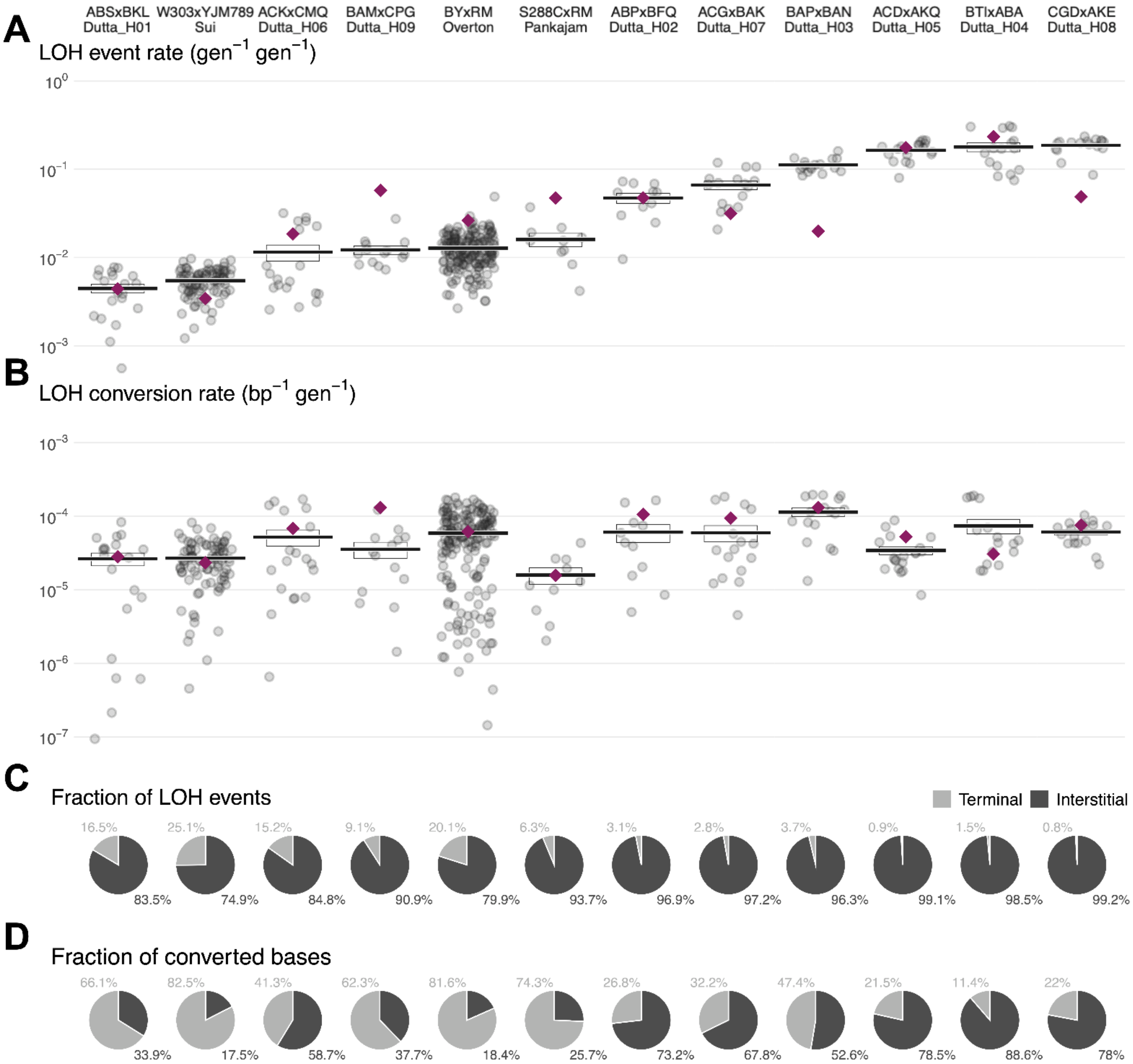

Figure S9. Overall LOH event and conversion rates. A. LOH event rates reported previously (purple diamonds) and revised using our method (after correction for undetected events). **B.** Same as A but for LOH conversion rates. **C.** Fractions of iLOH and tLOH events obtained with our revised method. **D.** Same as C but for converted bases.

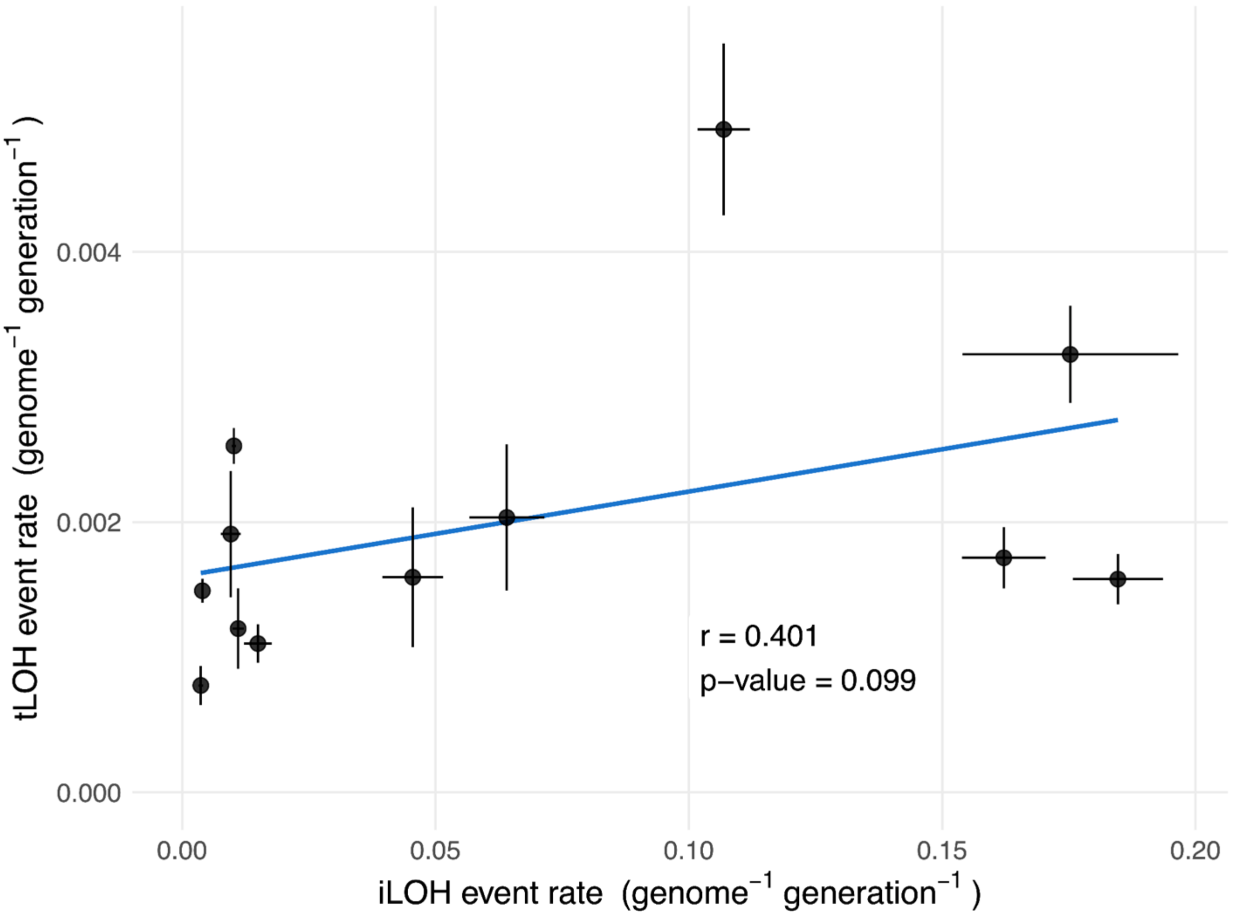

Figure S10. The rates of interstitial and terminal LOH events are uncorrelated. The Pearson correlation coefficient between the estimated iLOH and tLOH event rates across hybrids. P-value is obtained from a two-sided permutation test with Pearson's r as the test statistic.

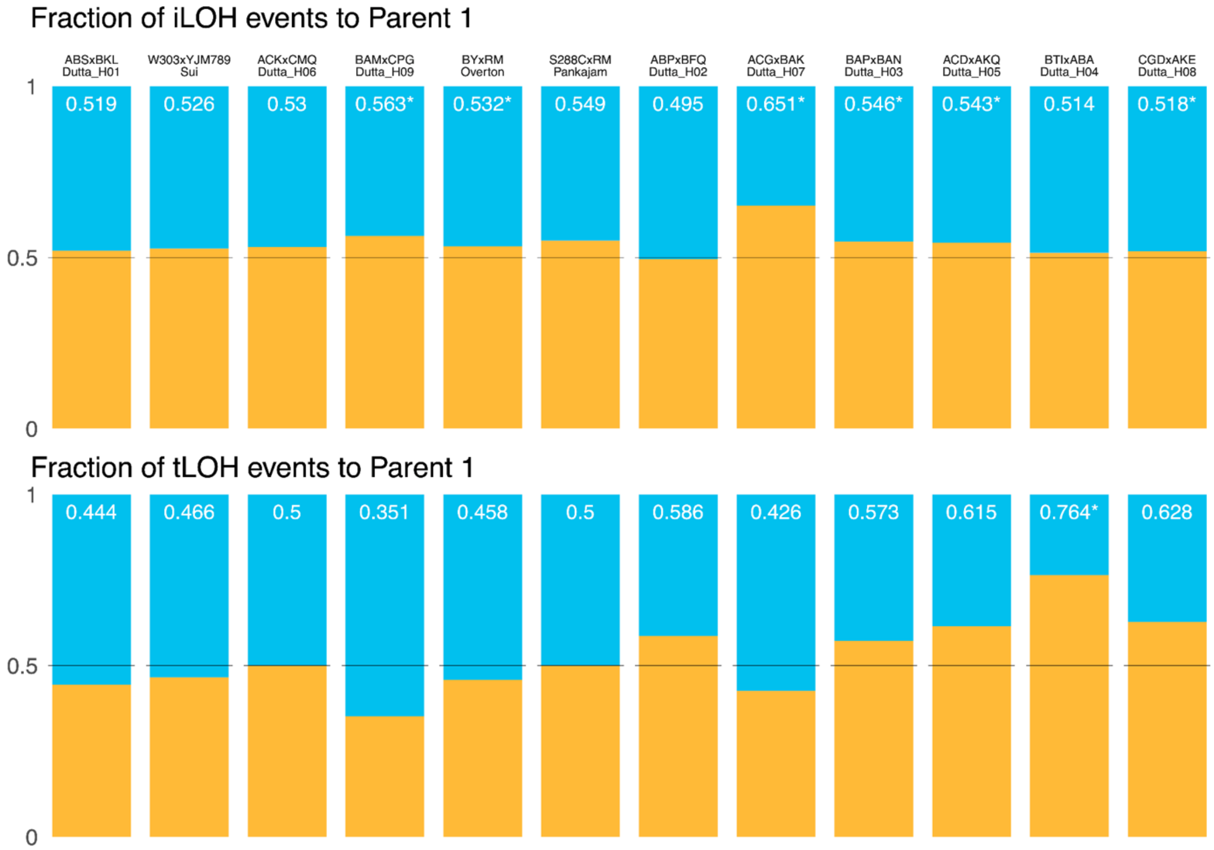

Figure S11. Homolog biases among iLOH and tLOH events. A. The fraction of iLOH events detected by the revised method that convert to each parent (color assignment to parents is arbitrary). Asterisk indicates statistical significance in the binomial test after Benjamini-Hochberg correction with FDR ≤ 0.05. **B.** Same as A but for tLOH events.

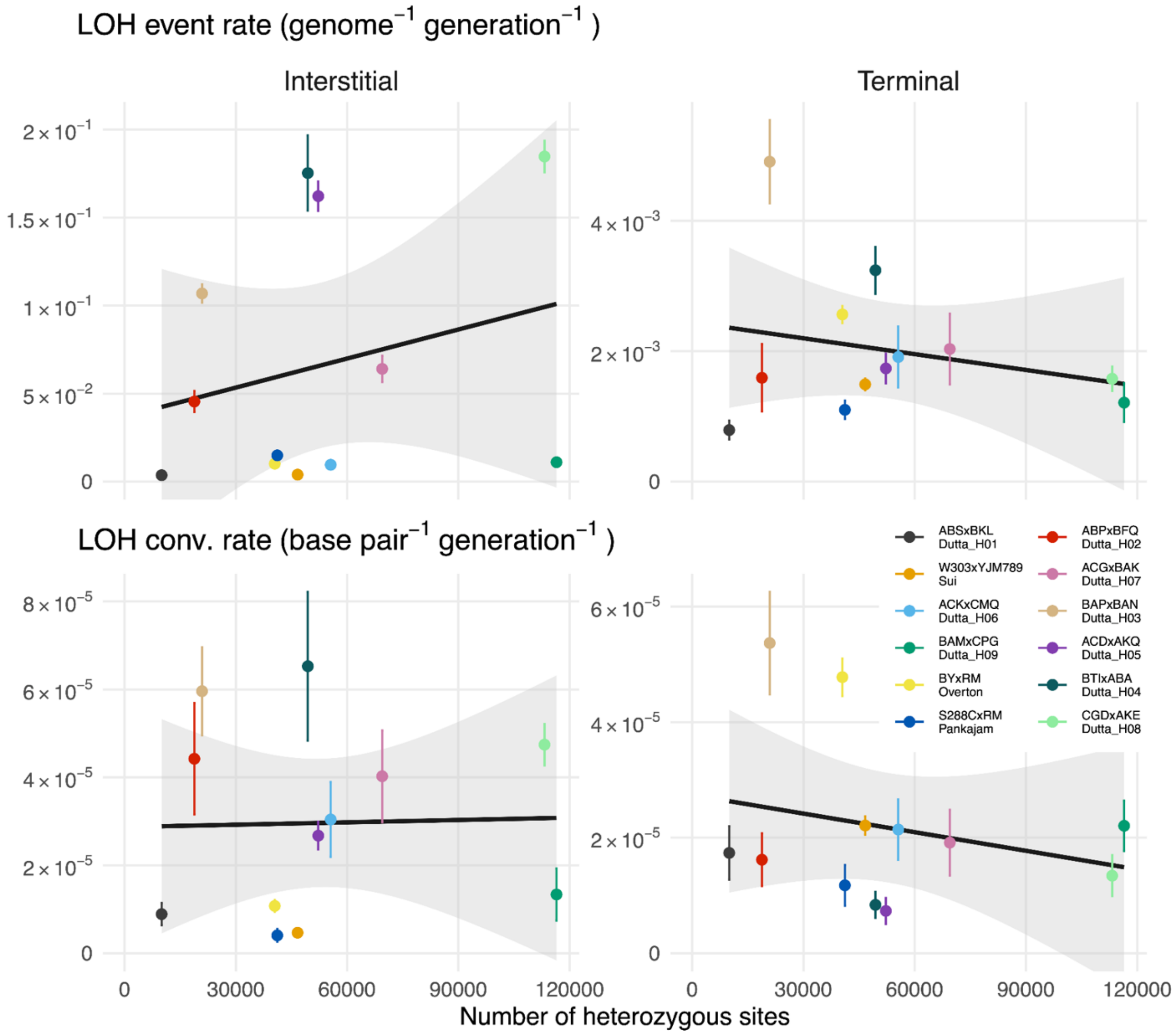

Figure S12. iLOH, tLOH event or conversion rates are not correlated with hybrid heterozygosity.
