# Supplementary material for: Improved genotype inference reveals cis- and trans-driven variation in the loss-of-heterozygosity rates in yeast
Source: Genetics. 2025 Dec 22;232(4):iyaf274. doi: 10.1093/genetics/iyaf274 (PMC13050209; doi:10.1093/genetics/iyaf274)
Supplement: iyaf274_Supplementary_Data [file iyaf274_supplementary_data.zip › Supplemental_Text_S1_GENETICS-2025-308599.pdf]

Supplementary Text for

Improved Genotype Inference Reveals Cis- and Trans-Driven Variation in the Loss-of-Heterozygosity Rates in Yeast

Michael S. Overton, Sergey Kryazhimskiy

Corresponding author: skryazhi@ucsd.edu

Calculation of the false heterozygote and false homozygote odds ratios

We would like to compare the genotype information from the end-point clones and the ancestor to identify sites where the founder genotype is erroneous. Since the true genotypes are unknown, here we develop a model for estimating the posterior odds ratio $R_{\text{false hom}}$ that the ancestor is a false homozygote at a given site (i.e., it is a heterozygote erroneously genotyped as a homozygote) and the posterior odds ratio $R_{\text{false het}}$ that the ancestor is a false heterozygote at a site (i.e., it is a homozygote erroneously genotyped as a heterozygote).

Model. Denote by A the number of copies of the derived allele in the diploid founder at a given site, such that $A \in \{0, 1\}$ with $A = 0$ denoting the homozygote for the ancestral allele and $A = 1$ denoting the heterozygote. Similarly D_i is the state of the ith end-point clone descendent from this founder, and $D_i \in \{0, 1, 2\}$ with $D = 2$ being the homozygote for the derived allele. Since neither A nor D_i are observable, we will treat them as random variables. Instead, we observe the inferred genotypes A' and D_i', with the observation probabilities defined as follows.

$$
\begin{aligned}
\Pr(X' = 2|X = 0) = \Pr(X' = 0|X = 2) &= 0, \\
\Pr(X' = 1|X = 0) = \Pr(X' = 1|X = 2) &= \varepsilon_{\text{HET}}, \\
\Pr(X' = 0|X = 1) = \Pr(X' = 2|X = 1) &= \varepsilon_{\text{HOM}}, \\
\Pr(X' = 0|X = 0) = \Pr(X' = 2|X = 2) &= 1 - \varepsilon_{\text{HET}}, \\
\Pr(X' = 1|X = 1) &= 1 - 2\varepsilon_{\text{HOM}},
\end{aligned}
$$

where X stands for either A or D_i and X' stands for either A' or D_i', respectively; ε_{HOM} is the false homozyte probability and ε_{HET} is the false heterozygote probability. We assume that both $\varepsilon_{\text{HOM}} \ll 1$ and $\varepsilon_{\text{HET}} \ll 1$.

During the mutation accumulation experiment, the state of the site can change from its ancestral values A to its end-point value D_i due to two biological processes.

1. An LOH event may occur in the vicinity of the focal site with probability λ. In this case, if the site was initially heterozyous ($A = 1$), it will be converted to either the ancestral ($D_i = 0$) or derived ($D_i = 2$) homozygous state with probability 1/2.

2. A mutation may occur at the focal site. Approximately two thirds of new mutations create a new allele (i.e., an allele that is neither ancestral nor the derived one). However, since we discard sites that have more than two alleles, we ignore this possibility. The remaining approximately one third of mutations flip the ancestral allele to the derived one or vice versa. Suppose that such mutations occur with probability μ. If the site is initially homozygous ($A = 0$), such a mutation will convert it to a heterozygous state ($D_i = 1$). If the sites is initially heterozygous ($A = 1$), such a mutation will convert it to either the ancestral ($D_i = 0$) or derived ($D_i = 1$) homozygous state with probability 1/2.

Thus, we have

$$
\begin{aligned}
\Pr(D_i = 0|A = 0) = \Pr(D_i = 2|A = 2) &= 1 - \mu, \\
\Pr(D_i = 1|A = 0) = \Pr(D_i = 1|A = 2) &= \mu, \\
\Pr(D_i = 2|A = 0) = \Pr(D_i = 0|A = 2) &= 0, \\
\Pr(D = 0|A = 1) = \Pr(D = 2|A = 1) &= \frac{\lambda + \mu}{2}, \\
\Pr(D = 1|A = 1) &= 1 - \lambda - \mu.
\end{aligned}
$$

False homozygote odds ratio. Suppose that among n descendent end-point clones, we have K_0, K_1 and K_2 that have the observed state 0, 1, and 2, respectively (with $K_0 + K_1 + K_2 = n$). Given this configuration of observed genotypes of end-point clones, the posterior odds ratio that the site in question is a false homozygote is

$$
\begin{aligned}
R_{\text{false hom}} &= \frac{\Pr(A = 1 \,|\, A' = 0, K_0, K_1, K_2)}{\Pr(A = 0 \,|\, A' = 0, K_0, K_1, K_2)} \\
&= \frac{\Pr(A' = 0, K_0, K_1, K_2 \,|\, A = 1)\ \Pr(A = 1)}{\Pr(A' = 0, K_0, K_1, K_2 \,|\, A = 0)\ \Pr(A = 0)}. \qquad \text{(S1)}
\end{aligned}
$$

Conditional on the state of the founder being $A = 1$, all descendants and the observation of the founder are independent, which implies that the configuration of the descendents

(K_0, K_1, K_2) is a multinomial random variable with the number trials n and the probabilities of outcomes p_{10}, p_{11} and p_{12}, where

$$p_{10} = p_{12} = (1-\lambda-\mu)\,\varepsilon_{\mathrm{HOM}} + \frac{\lambda+\mu}{2}\,(1-\varepsilon_{\mathrm{HET}})\,, \tag{S2}$$
$$p_{11} = (1-\lambda-\mu)\,(1-2\varepsilon_{\mathrm{HOM}}) + (\lambda+\mu)\,\varepsilon_{\mathrm{HET}} \tag{S3}$$

are the probabilities of observing an end-point clone in state 0, 1 and 2, given that the true state of the founder is 1. Therefore, we have

$$\begin{aligned}\Pr(A'=0, K_0, K_1, K_2 \,|\, A=1) &= \varepsilon_{\mathrm{HOM}}\, P_{\mathrm{mult}}(K_0, K_1, K_2; n, p_{10}, p_{11}, p_{12}) \\ &= \varepsilon_{\mathrm{HOM}}\, \frac{n!}{K_0!K_1!K_2!}\, p_{10}^{K_0}\, p_{11}^{K_1}\, p_{12}^{K_2}. \end{aligned} \tag{S4}$$

Analogously, we obtain

$$\Pr(A'=0, K_0, K_1, K_2 \,|\, A=0) = (1-\varepsilon_{\mathrm{HET}})\; P_{\mathrm{mult}}(K_0, K_1, K_2; n, p_{00}, p_{01}, p_{02}), \tag{S5}$$

where

$$p_{00} = (1-\mu)\,(1-\varepsilon_{\mathrm{HET}}) + \mu\,\varepsilon_{\mathrm{HOM}}, \tag{S6}$$
$$p_{01} = (1-\mu)\,\varepsilon_{\mathrm{HET}} + \mu\,(1-2\varepsilon_{\mathrm{HOM}})\,, \tag{S7}$$
$$p_{02} = \mu\,\varepsilon_{\mathrm{HOM}}. \tag{S8}$$

are the probabilities of observing an end-point clone in state 0, 1 and 2, given that the true state of the founder is 0.

Finally, we denote the prior probability for the ancestor to be a homozygote as $\Pr(A=0) = f$ and therefore $\Pr(A=1) = 1-f$. Then, substituting expressions (S4) and (S5) into equation (S1), we obtain

$$R_{\mathrm{false\,hom}} = \frac{\varepsilon_{\mathrm{HOM}}}{1-\varepsilon_{\mathrm{HET}}}\,\frac{1-f}{f}\,\left(\frac{p_{10}}{p_{00}}\right)^{K_0}\left(\frac{p_{11}}{p_{01}}\right)^{K_1}\left(\frac{p_{12}}{p_{02}}\right)^{K_2}. \tag{S9}$$

False heteozygote odds ratio. We calculate the false heterozgote odds ratio analogously.

$$\begin{aligned} R_{\mathrm{false\,het}} &= \frac{\Pr(A=0\,|\,A'=1, K_0, K_1, K_2)}{\Pr(A=1\,|\,A'=1, K_0, K_1, K_2)} \\ &= \frac{\Pr(A'=1, K_0, K_1, K_2\,|\,A=0)\;\Pr(A=0)}{\Pr(A'=1, K_0, K_1, K_2\,|\,A=1)\;\Pr(A=1)}. \end{aligned}$$

Following the same logic as above, we have

$$\begin{aligned}\Pr(A'=1, K_0, K_1, K_2 \,|\, A=0) &= \varepsilon_{\mathrm{HET}}\, P_{\mathrm{mult}}(K_0, K_1, K_2; n, p_{00}, p_{01}, p_{02}), \\ \Pr(A'=1, K_0, K_1, K_2 \,|\, A=1) &= (1-2\,\varepsilon_{\mathrm{HOM}})\; P_{\mathrm{mult}}(K_0, K_1, K_2; n, p_{10}, p_{11}, p_{12}),\end{aligned}$$

which yields

$$R_{\mathrm{false\,het}} = \frac{\varepsilon_{\mathrm{HET}}}{1-2\,\varepsilon_{\mathrm{HOM}}}\,\frac{f}{1-f}\,\left(\frac{p_{00}}{p_{10}}\right)^{K_0}\,\left(\frac{p_{01}}{p_{11}}\right)^{K_1}\,\left(\frac{p_{02}}{p_{12}}\right)^{K_2}$$
$$= \frac{\varepsilon_{\mathrm{HET}}}{1-2\,\varepsilon_{\mathrm{HOM}}}\,\frac{\varepsilon_{\mathrm{HOM}}}{1-\varepsilon_{\mathrm{HET}}}\,\frac{1}{R_{\mathrm{false\,hom}}}. \tag{S10}$$
